# Preventive effect of a high fluoride toothpaste and arginine-carbonate toothpaste on dentinal tubules exposure followed by acid challenge: a dentine permeability evaluation

**DOI:** 10.1186/1756-0500-7-385

**Published:** 2014-06-24

**Authors:** Shelon Cristina Souza Pinto, Matheus Coelho Bandéca, Michele Carolina Pinheiro, Rodrigo Cavassim, Mateus Rodrigues Tonetto, Alvaro Henrique Borges, José Eduardo Cezar Sampaio

**Affiliations:** 1 Department of Dentistry, Ponta Grossa State University, Ponta Grossa, PR, Brazil; 2 Department of Post-Graduate Program in Dentistry, CEUMA University, Sao Luis, MA, Brazil; 3 Department of Dentistry, Araraquara Dental School, São Paulo State University, Araraquara, SP, Brazil; 4 Department of Post-Graduate Program in Integrated Dental Sciences, University of Cuiabá, Cuiabá, MT, Brazil

**Keywords:** Tooth erosion, Dentin permeability, Toothpastes

## Abstract

**Background:**

Considering the current high use of high fluoride toothpastes, the aim of the study was to quantify alterations in the root dentine permeability submitted to treatment with a high fluoride toothpaste and 8% arginine, calcium carbonate, sodium monofluorophosphate toothpaste as a preventive treatment for dentinal tubules exposure followed by acid challenge.

**Methods:**

Thirty-third molars were sectioned below the cementoenamel. The root segments were connected to a hydraulic pressure apparatus to measure dentine permeability after the following sequential steps (n = 10 per group): I) Baseline; II) treatment with phosphoric acid for 30 s (maximum permeability); III) Toothbrushing (1 min) according to the experimental groups (G1- control; G2- 5000 ppm fluoride toothpaste; G3- 8% arginine-calcium carbonate toothpaste); IV) acid challenge for 5 min (orange juice). The data were converted into percentage, considering stage II as 100%.

**Results:**

The results have shown a statistically significant decreasing on dentine permeability after treatment with toothpaste (Friedman test and Dunn’s post hoc test). Comparison among groups demonstrated a high increasing on dentine permeability when acid challenge was performed after toothbrushing with distilled water (control group) (Kruskal-Wallis and Dunn’s post hoc test).

**Conclusion:**

The toothpaste treatment may provide sufficient resistance on dentine surface, preventing dentinal tubules exposure after acid challenge.

## Background

Dentine hypersensitivity (DH) is one of the most common problems in clinical practice [1,2]. Epidemiological studies suggest that the prevalence of DH is increasing [3-5]. Thus, an effective control of the etiological factors has not been achieved.

There are many etiologic and predisposing factors related to DH [6]. Enamel removal may be a result of attrition, abrasion and erosion. Root surface denudation is a result of cementum and periodontal tissue loss [7]. Root area exposure may be multifactorial, resulting of chronic trauma from toothbrushing, oclusal trauma, periodontal diseases and acid diet [8].

The consumption of acidic soft drinks has been shown an increase worldwide [8,9]. Acid challenges are able to remove tooth structure, leading to dentinal tubules exposure. It has been clearly shown that opened dentinal tubules, dentine permeability and dentine hypersensitivity are in close relationship [10,11].

Fluoride has been tested as a therapeutic approach protecting dental tissues from an acid challenge. Fluorappatite is able to reduce enamel and dentine solubility [12]. In order to provide a preventive effect against acid challenges, a daily base treatment would be an ideal approach [9]. Therefore, a high fluoride concentration toothpaste may reduce tooth wear, avoiding dentinal tubules exposure [13].

A new innovative toothpaste based upon 8% arginine, calcium carbonate, and 1450 ppm fluoride as sodium monofluorophosphate has been validated as a highly effective treatment for dentine hypersensitivity [14,15]. The precipitation of calcium- and phosphate-containing material on the tooth surface has demonstrated to be acid resistant after several expositions to cola drink [15].

Different composition of toothpastes have been constantly introduced, which may have important functions in preventing the loss of tooth structure and exposure of dentinal tubules followed by acid challenge. Thus, the aim of the present *in vitro* study was to quantify alterations in the root dentine permeability submitted to treatment with a high fluoride toothpaste (Duraphat**®**5000 ppm Fluoride Toothpaste) and 8% arginine, calcium carbonate, sodium monofluorophosphate toothpaste (Colgate Sensitive Pro-Relief ®) as a preventive treatment for dentinal tubules exposure followed by acid challenge.

## Methods

This study was approved by the Research Ethics Committee of Araraquara Dental School, UNESP (#52/04).

### Specimens preparation

Thirty human third molars extracted for surgical reasons from young patients (18 to 23 years old) previously stored in normal saline were used in the study. Two parallel grooves 0.5 mm deep on the root surface were performed: one at the cementoenamel junction and another 4 mm apical to the first. The area between the two grooves was flattened [10,16] and crowns were sectioned.

**Figure 1:**
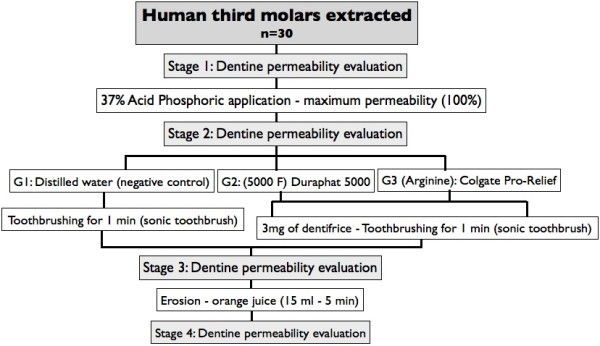
Schematic presentation showing how specimens were created and how fluid permeability was measured.

The pulpal tissue was removed with a Hedström file. The root segment was glued with cyanoacrylate adhesive into a resin acrylic apparatus, which permits the perfusion of the connected pressure fluid-filled system (Figure 1) [10].

### Experimental groups

**Figure 2:**
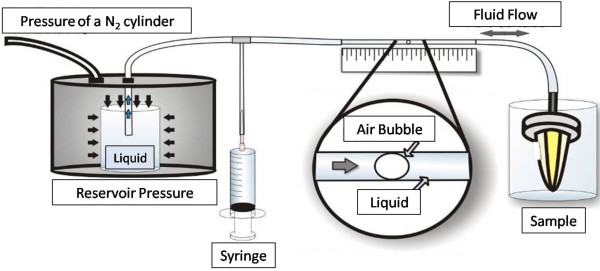
**Experimental design.** The specimens were divided into 3 groups (G1- Negative control; G2 – Duraphat® 5000 toothpaste; G3 – Colgate® Pro-Relief. Dentine permeability evaluation was performed at baseline (Stage 1); after 37% phosphoric acid application (Stage 2); after treatment (Experimental Groups- Stage 3); after erosion (Stage 4).

The specimens were randomly assigned into 3 groups (n = 10), according to the treatment (Figure 2):

– Group 1 (Negative Control): Distilled water.

– Group 2 (5000 F): Duraphat® 5000 toothpaste (Colgate Palmolive – 5000 ppm Fluoride).

– Group 3 (Arginine): Colgate Pro-Relief® (Colgate Palmolive – 8% arginine, calcium carbonate).

The treatment was performed following the manufacturer instructions. The exposed dentine area was brushed with 3 mg of toothpaste for 1 min (brushed carefully). Duraphat® 5000 toothpaste contains 5 mg of fluoride in 3 mg of toothpaste [17]. For the control group, the dentine exposure area was brushed with distilled water for 1 min.

After treatment, the specimens were carefully rinsed with distilled water (15 ml).

### Erosion cycle

Specimens were kept in a commercially available orange juice (Laranja caseira, Minute Maid Mais, Coca Cola®, SP, Brazil) (pH 3.80 ± 0.04). The orange juice (15 ml) was gently stirred (Fisher Scientific®) for 5 minutes. The specimens were removed from the orange juice and carefully rinsed with 15 ml of distilled water to remove acid excess from the surface.

The orange juice pH was measured at room temperature before each dentine permeability evaluation.

### Dentine permeability evaluation

In order to analyze dentine permeability, the root segment was connected to a fluid-filled system working at a pressure of 10 psi. A microcapillary tube with 25 μL diameter and length of 65 mm was positioned between the pressure reservoir and the root segment (Figure 1). Hydraulic conductance was measured through the length and diameter of microcapillary tube [10,11,18].

Each specimen was connected to the hydraulic pressure apparatus in order to measure root dentine permeability after the following sequential stages:

Stage 1: Baseline. Dentine permeability was measured before acid conditioning in order to observe if the phosphoric acid was able to open tubules.

Stage 2: Topical application of 37% phosphoric acid on dentine surface for 30 s in order to obtain the maximum permeability (100%) followed by rinsing with distilled water.

Stage 3: Toothbrushing with sonic toothbrush (Colgate Palmolive 360°, Colgate Palmolive, SP, Brazil) according to the experimental groups.

Stage 4: specimens were immersed in 15 mL of orange juice (Minute Maid Original, The Coca-Cola Company, Sao Paulo, SP, Brazil), for 5 min and rinsed with distilled water.

The hydraulic conductance was expressed as a percentage of the maximum value for each specimen. Therefore, each specimen was taken as its own control in order to analyze the preventive effect of toothpaste followed by acid challenge.

### Statistical analysis

The linear displacement of the air blister in the microcapillary tube was measured, according to the time unit, in each stage. The most prevalent value was used to calculate the fluid flow through dentine. The flow obtained after stage II was considered the maximum permeability (100%), and the other values were expressed as a percentage of the maximum. Data were analyzed using GraphPad Prism 5 statistical software (GraphPad La jolla, CA, USA). Level of significance was set at α = 0.05 (two-sided). Normal distribution was tested using the “D’Agostino & Pearson normality test”. Once the data did not showed normal distribution, means and standard deviations of permeability values were calculated and compared using non-parametric analysis. Friedman test and Dunn’s post hoc test were applied to evaluate the differences in permeability values after each stage into the same group and Kruskal-Wallis test and Dunn’s post hoc test were applied to evaluate the different stages among the groups.

## Results

The analysis results have been shown in the tables below. Table 1 shows the values of permeability distribution among groups.

**Table 1:** Mean values (%) and standard deviations of dentin permeability for the evaluated groups and stages

**Group**	**Stage**	**Mean (SD)**
Duraphat®	1	53.00 (11.00)
	2	100.00 (0.00)
	3	65.00 (20.00)
	4	65.00 (20.00)
ProArgin®	1	52.00 (16.00)
	2	100.00 (0.00)
	3	31.00 (16.00)
	4	31.00 (16.00)
Control	1	46.00 (16.00)
	2	100.00 (0.00)
	3	110.00 (22.00)
	4	180.00 (38.00)

Table 2 is demonstrating the comparison among stages.

**Table 2:** Comparison of the experimental stages within the same group and among the different groups

		**Duraphat**				**ProArgin**				**Control**			
		**S1**	**S2**	**S3**	**S4**	**S1**	**S2**	**S3**	**S4**	**S1**	**S2**	**S3**	**S4**
Duraphat®	S1		**										
	S2			*	*								
	S3							*					
	S4												*
ProArgin®	S1												
	S2							***	***				
	S3			*								***	
	S4												***
Control	S1											*	***
	S2												*
	S3							***					
	S4				*				***				

Evaluation of the differences in permeability values after each stage into the same group -Friedman and Dunn’s posttest - * = p < 0.05, *** = p < 0.001.

Evaluation of the different stages among the groups - Kruskal-Wallis and Dunn’s post-test - * = p < 0.05, ** = p < 0.01 and *** = p < 0.001.

## Discussion

The most common etiological factor related to dentine exposure followed by dentine hypersensitivity is soft drinks intake [5]. Soft drinks are able to cause tooth wear due to different acid features. There are some chemical aspects that can modulate the erosive potential of the soft drinks, such as, pH, titratable acidity, type of acid, buffer capacity, chelating properties and concentration of calcium, phosphates and fluoride [10,19].

Studies have been shown increasing on dentine permeability after acid drinks exposure [10,20]. However, preventive treatments are able to avoid loss of tooth structure. According to Hooper et al. [9], toothbrushing with toothpaste before meals may provide significant erosion protection in susceptible individual.

The method used for evaluating dentine permeability in the present study was hydraulic conductance. Hydraulic conductance is able to analyze qualitatively and quantitatively dentine permeability, since it allows the evaluation of presence of particles into the tubules [20]. Dentine permeability is evaluated by calculating the hydraulic conductance (Lp) by method of fluid filtration [21]. A constant hydrostatic pressure of 10 psi was chosen. This methodology has been used in other studies [10,20].

For the first stage, hydraulic conductance was evaluated in order to observe if stage 2 was able to open dentinal tubules. At stage 2, acid conditioning of the dentine exposure was performed and this value was considered 100%. Thus, the changes in permeability for the following stages were analyzed as a percentage of Stage 2 [10,20]. After acid conditioning (Stage 2), specimens were treated (Stage 3) followed by acid exposure (Stage 4). Dentine permeability measurements were performed after each stage.

This study has demonstrated higher dentine permeability when samples were brushed with distilled water and kept in orange juice (control group). The specimens were kept for 5 min on soft drink, considering that it is the time necessary for acid neutralization and/or removal on tooth surfaces by saliva [22]. Prior studies have been demonstrating that the application of acid drinks for a relatively short exposure of time is sufficient to induce changes on dentine surface [10,23,24]. Orange juice was able to increase the permeability after a 5-min single application and may perfectly simulate the typical oral assumption that occurs several times a day [10]. The erosive potential of orange juice may be justified due to the low pH (3.80 ± 0.04), type of acid (acid citric), titratable acidity (1.23 ± 0.08) and high capacity to hydroxyapatite dissolution [19]. Citric acid is also one of the most erosive due to their quelling capacity, which is responsible by calcium seize from saliva and teeth [25].

Nevertheless, the results showed a greater reduction of permeability when specimens were brushed with toothpaste before acid challenge. Therefore, toothpastes may present a preventive effect in the dentinal tubules exposure. Toothpaste can be an important type of treatment for various dental problems, since it is easily accessible for the population. A high fluoride and an arginine-calcium carbonate toothpaste were tested and its ability as a preventive treatment to dentinal tubules exposure. Several studies have been shown that toothbrushing with toothpaste is able to occlude dentinal tubules or decreasing dentine permeability, due to the presence of desensitizing agents or abrasives particles in tubules [15,26,27].

According to Diamanti et al. [13], higher preventive effect of dentine erosion can be reached when 5000 ppm Fluoride toothpaste is applied. Fluoride application is able to increase dentine’s resistance to erosive challenge [13,28] through surface adsorption and hetero-ionic exchange with surface hydroxyl ions [13], if it is in sufficient concentration.

The results have been shown a preventive effect of Duraphat® 5000 Fluoride for acid challenge. The same effect was also showed by arginine-calcium carbonate toothpaste, which demonstrated occlusion of dentinal tubules and decreasing of dentine permeability after its application. Although, no significant differences have been shown between toothpaste, it cannot be concluded that both have the same effect, since the preventive effect may change after many acid challenges.

Petrou et al. [15] has clearly shown that the arginine associated with calcium carbonate is highly effective in occluding dentinal tubules and the occlusion has demonstrated to be resistant to acid challenges. It happens due to the following mechanism of action: arginine facilitates the adherence of calcium carbonate to the surface in a basic pH, and when arginine is associated to calcium carbonate triggers deposition of phosphate on dentine surface and within dentinal tubules [14,15].

Controversial results considering toothpaste treatment can be found when it is applied after acid challenge. According to previous studies [29,30], toothbrushing with toothpaste may cause dentine abrasion or erosion in a variable degree, resulting in tubule opening. However, other in vitro studies showed decreasing on dentine permeability when toothbrushing with toothpaste was performed after acid challenge [10,26].

In agreement with previous studies [13,27,31], this current study has demonstrated that toothpaste application is effective in increasing dentine’s resistance to erosive challenge. However, more than one cycle of erosion must be tested in order to observe the preventive effect of toothpastes after many acid challenges.

Considering the methodology employed in this study, and based upon its limitations, it can be concluded that toothbrushing associated with a high fluoride or arginine-calcium carbonate toothpaste is able to prevent tooth wear and dentinal tubules exposure in vitro. Thus, clinicians must advise patients with risk of erosion or abrasion to brush their teeth before meals and avoid acid drinks intake.

## Conclusion

Considering the methodology employed in this study, and based upon its limitations, it can be concluded that toothbrushing associated with a high fluoride or arginine-calcium carbonate toothpaste is able to prevent tooth wear and dentinal tubules exposure in vitro. Thus, clinicians must advise patients with risk of erosion or abrasion to brush their teeth before meals and avoid acid drinks intake.

## Competing interests

The authors declare that they have no competing interests.

## Authors’ contributions

All authors have made substantial contributions for elaborate this article as following: SCSP participated in drafting the article, selection and execution of the methodology; MCB participated in drafting the article, elaboration of the experimental design and the execution of the study. MCP participated the execution of the study and revised the article. RC participated in drafting the article and execution of the study. MRT participated the execution of the study and revised the article. AHB participated in drafting the article and execution of the study. JECS revised the article critically for important intellectual content, elaboration of the study design and interpretation of data. All authors read and approved the final manuscript.
